# Viral and Bacterial Respiratory Pathogens during the COVID-19 Pandemic in Israel

**DOI:** 10.3390/microorganisms11010166

**Published:** 2023-01-09

**Authors:** Yonatan Oster, Wiessam Abu Ahmad, Ayelet Michael-Gayego, Mila Rivkin, Leonid Levinzon, Dana Wolf, Ran Nir-Paz, Hila Elinav

**Affiliations:** 1 Department of Clinical Microbiology and Infectious Diseases, Hadassah Hebrew University Medical Center, Jerusalem 9112001, Israel; 2 The Faculty of Medicine, Hebrew University of Jerusalem, Jerusalem 9190401, Israel; 3 Braun School of Public Health and Community Medicine, Hebrew University of Jerusalem, Jerusalem 9190401, Israel; 4 Clinical Virology Unit, Hadassah Hebrew University Medical Center, Jerusalem 91120, Israel

**Keywords:** SARS-CoV-2, respiratory pathogens, viral, bacterial

## Abstract

Background: previous worldwide reports indicated a substantial short-term reduction in various respiratory infections during the early phase of the SARS-CoV-2 pandemic. Aims: exploring the long-term impact of the COVID-19 pandemic on respiratory pathogens. Methods: retrospective analysis of bacterial and viral positivity rate in respiratory samples, between 1 January 2017–30 June 2022 in a tertiary hospital in Jerusalem, Israel. Results: A decline in overall respiratory tests and positivity rate was observed in the first months of the pandemic. Respiratory isolations of *Hemophilus influenza* and *Streptococcus pneumoniae* were insignificantly affected and returned to their monthly average by November 2020, despite a parallel surge in COVID-19 activity, while *Mycoplasma pneumoniae* was almost eliminated from the respiratory pathogens scene. Each viral pathogen acted differently, with adenovirus affected only for few months. Human-metapneumovirus and respiratory-syncytial-virus had reduced activity for approximately a year, and influenza A virus resurged in November 2021 with the elimination of Influenza-B. Conclusions: After an immediate decline in non-SARS-CoV-2 respiratory infections, each pathogen has a different pattern during a 2-year follow-up. These patterns might be influenced by intrinsic factors of each pathogen and different risk reduction behaviors of the population. Since some of these measures will remain in the following years, we cannot predict the timing of return to pre-COVID-19 normalcy.

## 1. Introduction

SARS-CoV-2, the pathogen causing COVID-19 disease, first identified in December 2019 in Wuhan, China, has since spread globally and was declared as a pandemic in March 2020 by the World Health Organization [1]. This ongoing pandemic features enormous medical, economic, and social implications on human lives that span beyond the infection itself. In the first months of COVID-19 spread, until vaccines became available by the end of 2020, the main strategies utilized in combating COVID-19 included the endorsement of the use of facial masks and social distancing, coupled, in some countries, with the implementation of periods of lockdown and school closures. Former reports indicated that the high public compliance with these restrictions, noted during these first months of the COVID-19 pandemic, was associated with a marked reduction in the incidence of other respiratory pathogens. The COVID-19 outbreak was delayed in Israel, as the first case was reported on 27 February 2020, with a gradual response by the health authorities and lockdown employed only on 17 March 2020. Previously, we reported a trend of a 46% to 100% reduction in the prevalence rate of viral pathogens as well as *Mycoplasma pneumoniae* and *Bordetella pertussis* in April–August 2020, including in adults and pediatric cases [2]. Similarly, Kuitinen reported a 2.7-and-3.6-fold reduction in two pediatric emergency departments (PED) visits in Finland during March–April 2020 [3]. Angolulvant et al. found a 68% and 45% decline in PEDs visits and admissions due to communicational diseases during the same period that was the lockdown period in France [4]. Fredrich reported a significant reduction in acute bronchiolitis hospitalizations in Brazil [5], and a similar trend in respiratory-related admissions was reported in the US, with a recovery in the final weeks of 2020 [6]. The rapidly aborted and lower influenza activity was reported in Japan [7], Finland [3], Korea [8], Australia [9], and the northern hemisphere [10]. While most authors attributed this effect to restrictive measures and nonpharmaceutical interventions [11,12,13,14], some claimed [15,16] that this observation could result from interference and not only due to physical barriers and distancing. The prolongation of the pandemic, with the introduction of vaccines, resulted in lowered anxiety, coupled with public exhaustion, driving a marked reduction in public compliance towards these barrier and social distancing recommendations, thus enabling the exploration of this theory. In this study, we evaluate the incidence of major respiratory infections by both viral and bacterial pathogens over a more than two-year COVID-19 pandemic perspective, while assessing the implications of the shifting public compliance to COVID-19-related restrictions recommendations on non-COVID-19 respiratory morbidity.

## 2. Methods

The Hadassah-Hebrew University Medical Center in Jerusalem is a tertiary care medical center consisting of two hospitals with 1100 inpatient beds, constituting one of the two major medical centers in Jerusalem, together serving a population of about 1 million. Patients hospitalized at the Hadassah Medical Center with acute respiratory infection are routinely tested from 2014, for the following 7 (non-SARS-CoV-2) viruses (i.e., respiratory virus panel): influenza A(H1N1), A(H3N2) and B viruses, respiratory syncytial virus (RSV), human metapneumovirus (HMPV), parainfluenza viruses 1–3, and adenovirus, by multiplex RT-PCR as described [17]. Additionally, patients were tested by PCR for *Mycoplasma pneumoniae* [18] in accordance with clinical indications and treating physicians’ discretion. For data collection, we used the respiratory pathogens data specified above as well as data of respiratory bacterial pathogens isolated by sputum culture, in cases of suspected pneumonia accompanied by a productive cough, including *Haemophilus influenzae* and *Streptococcus pneumoniae*. We included all the tests performed at the Hadassah Medical center from January 2017 to June 2022. Duplicates of samples submitted to patients on the same date were excluded. We then compared this data to the number of respiratory samples received every month at the microbiology and the virology labs, as a proxy for the burden of respiratory patients in the hospital in that month. Of note, between 15 November 2017–31 December 2017, no samples for mycoplasma PCR were processed in our lab due to technical issues. In addition, we extracted numbers of new SARS-CoV-2 cases in Jerusalem from the Israeli Ministry of Health database as an indicator of COVID-19 activity. The binary segmentation algorithm was used for locating multiple change-points (i.e., time points when the series changes its behavior), setting the maximum number of 5 change points for each series. Analysis was done using R statistical software version 3.5.0 (R Project for Statistical Computing).

## 3. Results

**Figure 1:**
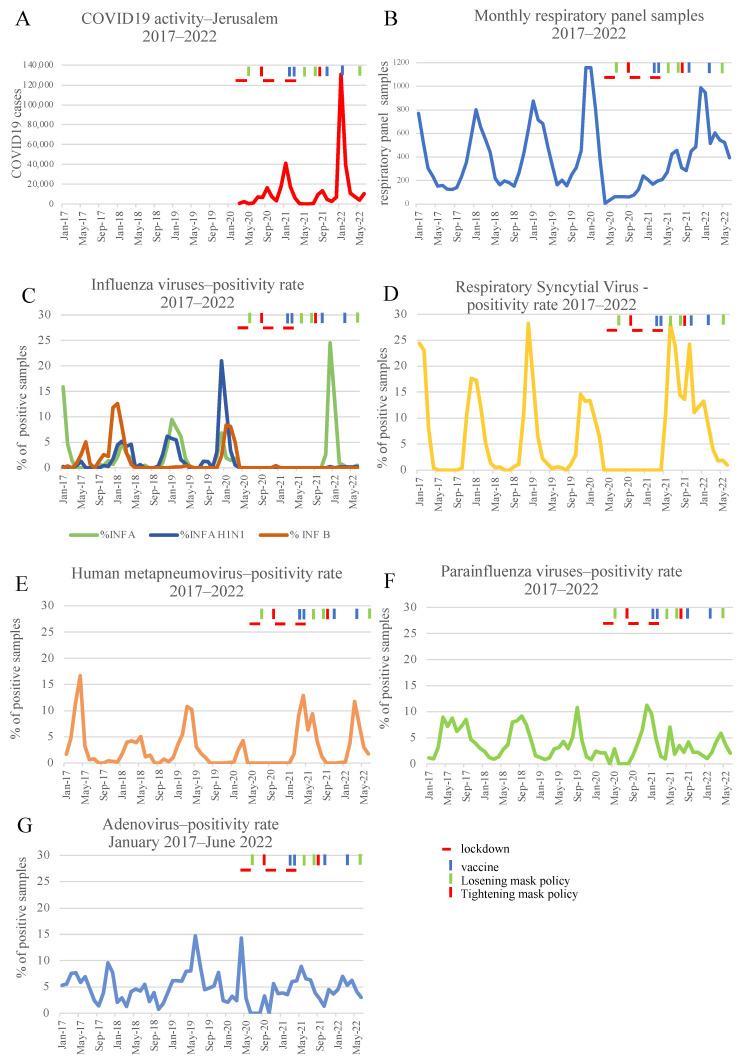
Viral pathogens in the pre-COVID-19 and during the COVID-19 pandemic. (**A**). COVID-19 activity-Monthly COVID-19 cases in Jerusalem district. (**B)**. Monthly viral samples submitted to the Hadassah Virology lab (January 2017–June 2022). (**C**). Monthly positivity rate (%) of influenza virus in samples submitted to the Hadassah Virology lab (January 2017–June 2022) green influenza A, blue H1N1, orange influenza B. (**D**). Monthly positivity rate (%) of respiratory syncytial virus (RSV) in samples submitted to the Hadassah Virology lab (January 2017–June 2022). (**E**). Monthly positivity rate (%) of human metapneumovirus (HMPV) in samples submitted to the Hadassah Virology lab (January 2017–June 2022). (**F**). Monthly positivity rate (%) of parainfluenza virus (combined 1–3) in samples submitted to the Hadassah Virology lab (January 2017–June 2022). (**G**). Monthly positivity rate (%) of adenovirus in samples submitted to the Hadassah Virology lab (January 2017–June 2022).

COVID-19 cases were first reported in the Jerusalem greater area in March 2020, and followed a waves and remission pattern described in Figure 1A, with peaks in April and September 2020, January and September 2021, and January 2022. Between January 2017 and June 2022, 25,225 respiratory samples were tested for respiratory viruses (other than SARS-CoV-2) at the Clinical Virology lab of the Hadassah Medical center, with a median of 307 samples per month. Each sample was tested for a panel of seven respiratory viruses (see Methods). During these years, December and January were the most active months with a pick of 1157 samples delivered to the lab in December 2019. In April 2020, there was steep drop in the number of samples that were tested with only 7 samples (reflecting the first wave of COVID-19 in Israel) in comparison with an average of 387 samples in April 2017, 2018, and 2019, Figure 1B). Only in March 2021, respiratory virus sampling returned to its former yearly seasonal variation. We next investigated the patten of each respiratory virus included in the respiratory panel, including the monthly number of positive samples (Figure S1 Supplementary) and monthly positivity rate (Figure 1). HMPV (Figure 1D) and RSV (Figure 1E) followed this trend, both eliminated in April 2020. HMPV re-emerged in February 2021, and according to our analysis, the trend of RSV positivity rate changed (decreased) in March 2000 and reappeared in April 2021. The effect upon the influenza virus was much more substantial and not related to sampling since there were no cases of influenza during the winter of 2020–2021. Only in November 2021, influenza A/H3N2 reappeared with almost no cases of influenza A/H1N1 or influenza B until the end of our survey in June 2022. Interestingly, both the parainfluenza virus (Figure 1F) and adenovirus (Figure 1G) circulation were only mildly affected during the pandemic waves. The adenovirus positivity rate decreased between April to October 2020, but ever since returned to its pre-COVID-19 pandemic pattern being monthly identified in 4.57 % (range 0–8.8%) out of the viral respiratory samples. No change in trend was identified by the binary segmentation algorithm of parainfluenza from April 2017 until February 2021, indicating a non-significant effect during this period.

We next explored whether the same effect presented in bacterial respiratory pathogens *Hemophilus Influenza, Pneumococcus pneumoniae,* and *Mycoplasma Pneumoniae.*

**Figure 2:**
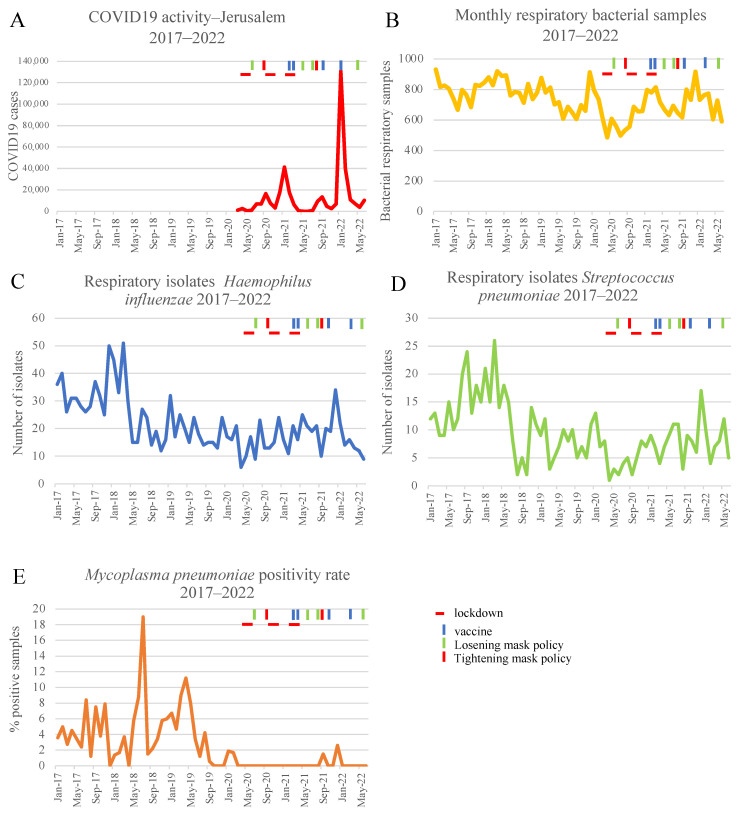
Bacterial pathogens in the pre-COVID-19 and during the COVID-19 pandemic. (**A**). COVID-19 activity−Monthly COVID-19 cases in Jerusalem district. (**B**). Monthly sputum samples submitted to the Hadassah microbiology lab (January 2017–June 2022). (**C**). Monthly number of isolates of *Haemophilus influenza* recovered from sputum samples submitted to Hadassah microbiology lab (January 2017–June 2022). (**D**). Monthly number of isolates of *Streptococcus pneumoniae* recovered from sputum samples submitted to the Hadassah microbiology lab (January 2017–June 2022). (**E**). Monthly positivity rate (%) of samples submitted for *Mycoplasma pneumoniae* PCR submitted to the Hadassah Virology lab (January 2017–June 2022).

Variation of the bacterial sputum samples submitted to the bacteriology was less prominent than viral respiratory sampling. From January 2017 to June 2022, 48,394 sputum samples were delivered to the bacteriology lab, with a median of 735 monthly samples. The highest number of sputum samples was in January 2017 with 931 samples and the lowest number was 485 samples, again in April 2020 (Figure 2A). Using the binary segmentation algorithm, a marked decline in sputum sampling was noticed between February 2020 and September 2020, when sampling began to recover, with a further increase in December 2020.

Despite the decline in the sputum samples (Figure 2B), the number of bacterial respiratory isolates of *Hemophilus influenza* and *Streptococcus pneumonia* was affected only for a shorter duration. During April–July 2020, a monthly average of 10.5 of *Hemophilus influenza* isolates were cultured from sputum specimens. This 2.38-fold reduction in comparison with an average of 25 monthly isolates from January 2017–March 2020, lasted shortly, since in August 2020, 23 isolates were cultured (Figure 2C). *Streptococcus pneumoniae* respiratory infections were affected for a slightly more prolonged period and started to gain back their activity in November 2020. During April–October 2020, there was a 3.59 reduction in the number of isolates from the sputum from an average of 11.3 monthly isolates to 3.14. In November 2020, *Streptococcus pneumoniae* reappeared with eight cases cultured from sputum specimen in our bacteriology laboratory (Figure 2D). These changes where so temporary and subtle that the binary segmentation algorithm did not capture any change in trends of both bacteria during this period.

In contrast, *Mycoplasma pneumoniae* was almost eliminated from the respiratory pathogens scene. Until March 2020, 125.6 samples were submitted monthly for PCR analysis of *Mycoplasma pneumoniae*, with an average positivity rate of 4.56% (pick of 18% in July 2018). The number of samples submitted to the lab dropped to 42 samples in April 2020, and only 5 cases of mycoplasma pneumoniae were identified ever since, despite an average of 69.5 samples processed per month (Figure 2E). Statistical analysis indicated a change in trend of positivity rate of *Mycoplasma pneumoniae* that did not recover yet.

## 4. Discussion

One of the interesting and unexpected aspects of the global COVID-19 pandemic is its effect on the epidemiology of other common viral and bacterial respiratory infections. In this study, we describe this epidemiology in a tertiary-care medical center in Jerusalem, Israel, from January 2017 to June 2022, using comprehensive laboratory data. A steep decrease was seen in the number of tests and the positivity rate in the first months of the pandemic for almost all pathogens, as we previously described [2]. Similar findings were identified in many other countries [3,4,7,8,9]. Notably, the recovery pattern of each pathogen was different; looking at the viral pathogens (Figure 1), RSV infections have surprisingly peaked during the summer of 2021, similarly reported in the UK, Korea, and Morocco [14,19,20], while parainfluenza was not affected and adenovirus returned to a seasonal pattern which is very similar to the pre-COVID-19 years, on October 2020. The influenza viruses have skipped the 2020–2021 winter season and emerged again only a year later. All positive cases were identified as influenza type A, which might be associated with the global disappearance of the B-Yamagata lineage that was reported following the pandemic [21]. Regarding the bacterial pathogens (Figure 2), the number of monthly isolates from sputum specimens of *Haemophilus influenza* and *Streptococcus pneumoniae* only mildly and not significantly changed during the study period, while the detection of *Mycoplasma pneumoniae* had become a rare event since January 2020, similar to a report in a global survey [22] that we took part in. The decrease in most respiratory pathogens during the early stage of the pandemic in Israel can be explained by interventions which were applied to decrease SARS-CoV-2 spread, such as social distancing, masking, closing of schools, airports and workplaces, and in some high-risk periods even total curfews. Another important mechanism suggested by Piret [15] and Kiseleva [16] is termed viral interference and includes the possible downregulation of receptors, high levels of expression of interferons and interferon (IFN)-stimulated genes that prevent sequential viral infection as well as possible cross-reactive antibodies. This mechanism might explain the marked different effects that we observed between viral and bacterial pathogens (*Haemophilus influenza* and *Streptococcus pneumoniae*) with bacterial pathogens being non-significantly, mildly, and shortly affected compared to the more prolonged and substantial effect noticed among viral pathogens. The discrepancy between the different viral-pathogen patterns can be explained by a dissimilar effect of innate immune components upon the infection and replication of diverse viruses as well as pathogen-specific escape mechanisms. The scientific evidence for this theory are mainly ex vivo and in vivo models and some epidemiological data of viral interference [23,24,25]. Nevertheless, this seems less plausible since we observed that for many pathogens, the pattern returned quite early to some kind of “epidemiological normality”, regardless of the high activity of SARS-CoV-2 during January 2022. Additionally, the epidemiological differences can be attributed to some epidemiological determinants that are related to a few of the pathogens. For example, in the case of *Mycoplasma pneumoniae*, a 3–4 year surge [22] was reported and it is possible that distancing resulted in breaking this pattern, and for that reason it is now hard to detect these pathogens in the Jerusalem area. Other issues may reflect the transmissibility of a few of the pathogens. Thus, the introduction of vaccination and reduction in masking and social distancing resulted in a regain of pre-pandemic patterns in those pathogens. However, it will be hard to explain the exact differences between the different pathogens. The interplay between viral ecology and prevention measures used by the population will shape the new “normality” of respiratory infections, as SARS-CoV-2 continues to be an important human pathogen despite the reduced virulence of the current Omicron variants.

The main limitation of our survey is the collection of data from a two-campus hospital in one city, Jerusalem, which is serviced by one additional hospital. Although it reflects the population in Jerusalem, it might not reflect the situation in other areas of Israel. Jerusalem is a mixture of different cultural populations (secular jews, ultraorthodox jews, Arabs) and each community has different socioeconomical characteristics and different compliance to the ministry of health recommendations regarding social distancing, school closure, facial masking, and vaccination [26]. The second limitation stems from the retrospective methodology of our study. During the pandemic, we could have missed SARS-CoV-2 co-infection with other pathogens as in most cases of SARS-CoV-2 positive PCR or antigen tests (during later period of the pandemic), a respiratory panel test was not performed.

In summary, the pandemic of SARS-CoV-2 affected the incidence of other respiratory pathogens mainly in the pre-vaccine period when compliance to restriction measurements was higher, probably due to fear and insecurity in the population. As the long-term effect is not uniform upon bacterial and viral pathogens’ positivity rate, we suggest that there are other factors, host related, or pathogen related, that are responsible for the differential effect upon the incidence of different pathogens. Due to the multiple factors determining this impact, and the inconstancy of the patterns, we cannot predict when each pathogen will return to its normal epidemiological behavior.

## Data Availability

Due to our institutional policy, data will be available upon request only.

## Supplementary Materials

The following supporting information can be downloaded at: https://www.mdpi.com/article/10.3390/microorganisms11010166/s1, Figure S1: Viral pathogens in the pre-COVID-19 and during the COVID-19 pandemic; (A) COVID-19 activity-Monthly COVID-19 cases in Jerusalem district; (B) Monthly viral samples submitted to the Hadassah Virology lab (January 2017–June 2022); (C) Monthly cases of Influenza virus in samples submitted to the Hadassah Virology lab (January 2017–June 2022) green influenza A, blue H1N1, orange influenza B; (D) Monthly cases of Respiratory Syncytial Virus (RSV) in samples submitted to the Hadassah Virology lab (January 2017–June 2022); (E) Monthly cases of Human metapneumovirus (HMPV) in samples submitted to the Hadassah Virology lab (January 2017–June 2022); (F) Monthly cases of Parainfluenza virus (combined 1–3) in samples submitted to the Hadassah Virology lab (January 2017–June 2022); (G) Monthly cases of Adenovirus in samples submitted to the Hadassah Virology lab (January 2017–June 2022).
