# Supplementary material for: Viral and Bacterial Respiratory Pathogens during the COVID-19 Pandemic in Israel
Source: Microorganisms. 2023 Jan 9;11(1):166. doi: 10.3390/microorganisms11010166 (PMC9864990; doi:10.3390/microorganisms11010166)
Supplement: Supplementary file 1 [file microorganisms-11-00166-s001.zip › microorganisms-2102129-supplementary.pdf]

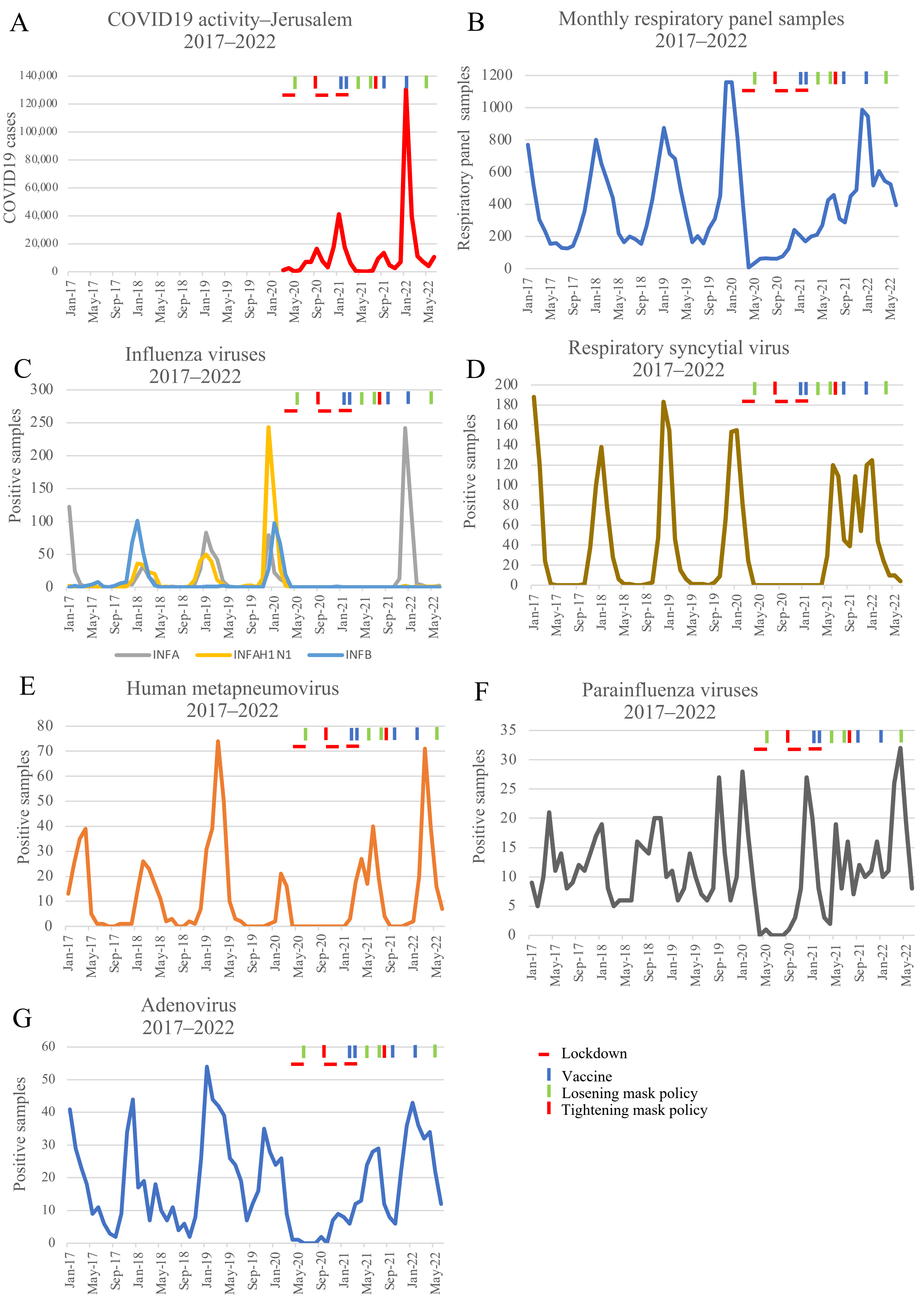

A
COVID19 activity–Jerusalem 2017–2022
COVID19 cases
B
Monthly respiratory panel samples 2017–2022
Respiratory panel samples
C
Influenza viruses 2017–2022
Positive samples
INFA
INFAH1N1
INFB
D
Respiratory syncytial virus 2017–2022
E
Human metapneumovirus 2017–2022
F
Parainfluenza viruses 2017–2022
G
Adenovirus 2017–2022
Lockdown
Vaccine
Losening mask policy
Tightening mask policy
